# Genomic Characterization of *mcr*-*1*-Carrying Foodborne *Salmonella enterica* serovar Typhimurium and Identification of a Transferable Plasmid Carrying *mcr*-*1*, *bla*_**CTX-M-14**_, *qnrS2*, and *oqxAB* Genes From Ready-to-Eat Pork Product in China

**DOI:** 10.3389/fmicb.2022.903268

**Published:** 2022-06-29

**Authors:** Lili Li, Xiulin Wan, Rikke Heidemann Olsen, Jian Xiao, Chong Wang, Xuebin Xu, Hecheng Meng, Lei Shi

**Affiliations:** ^1^Institute of Food Safety and Nutrition, Jinan University, Guangzhou, China; ^2^School of Food Science and Engineering, South China University of Technology, Guangzhou, China; ^3^Department of Veterinary and Animal Sciences, Faculty of Health and Medical Sciences, University of Copenhagen, Copenhagen, Denmark; ^4^Guangzhou Food Inspection Institute, Guangzhou, China; ^5^Shandong New Hope Liuhe Group Ltd., Qingdao, China; ^6^Department of Etiological Microbiology Laboratory, Shanghai Municipal Center for Disease Control and Prevention, Shanghai, China

**Keywords:** *S. typhimurium*, mcr-1, ESBLs, FQs, ready-to-eat pork product

## Abstract

*Salmonella enterica* resistant to colistin, third-generation cephalosporins (3GCs), and fluoroquinolones (FQs) has been deemed a high-priority pathogen by the World Health Organization (WHO). The objective of this study was to characterize 11 *mcr*-*1*-harboring *Salmonella enterica* serovar Typhimurium isolates from raw pork and ready-to-eat (RTE) pork products in Guangzhou, China. All isolates were multi-drug resistant and contained 6–24 antibiotic-resistant genes. The *mcr*-*1* gene was localized in the most conserved structure (*mcr*-*1*-*orf* ) in eight isolates and in mobile structure (IS*Apl1*-*mcr*-*1*-*orf* ) in three isolates. One raw pork isolate SH16SF0850, co-harbored *mcr*-*1*, *bla*_CTX−M−14_, and *oqxAB* genes. One isolate 17Sal008 carried *mcr*-*1*, *bla*_CTX−M−14_, *qnrS2*, and *oqxAB* genes located on a 298,622 bp IncHI2 plasmid pSal008, which was obtained from an RTE pork product for the first time. The pSal008 was closely related to a plasmid in an *S. typhimurium* isolate from a 1-year-old diarrheal outpatient in China and was found to be transferable to *Escherichia coli* J53 by conjugation. Genome sequence comparisons by core-genome Multi Locus Sequence Typing (cgMLST) based on all *S. typhimurium* isolates from China inferred highly probably epidemiological links between selected pork isolates and no possible epidemiologically links between RTE pork isolate 17Sal008 and other isolates. Our findings indicate that raw pork and pork products are potential reservoirs of *mcr-1*-harboring *S. typhimurium* and highlight the necessity for continuous monitoring of colistin, 3GCs, and FQs resistant *S. typhimurium* from different origins.

## Introduction

Strains of *Salmonella enterica* are a common concern in food safety, as they are the leading cause of global bacterial food poisoning outbreaks (Kirk et al., 2015). In 2017, the global estimate of non-typhoidal *salmonella* invasive disease cases reached 535,000 (GBD 2017 Non-Typhoidal *Salmonella* Invasive Disease Collaborators, 2019). In China, non-typhoidal *Salmonella* serovars are collectively the second most prevalent bacterial agents in cases of acute diarrhea, accounting for 25.1% of 59,384 cases, according to an epidemic study based on a national surveillance network for patients with acute diarrhea from 2009 to 2018 Wang et al. (2021).

*Salmonella enterica* serovar Typhimurium is one of the most frequently identified serovars among foodborne illnesses, livestock, and retail meat (particularly pork) (Zhang et al., 2014, 2016; Lu et al., 2019; Wu et al., 2021). The relevance of *S. typhimurium* is also marked by its capability to acquire resistance determinants to various drug classes, especially those of critical antibiotics, such as colistin, third-generation cephalosporins (3GCs), and fluoroquinolones (FQs), which may lead to clinical treatment failure (Yi et al., 2017; Lu et al., 2019).

The plasmid-encoded polymyxin resistance gene, *mcr-1*, was originally detected in *Enterobacteriaceae* from the environment, animals, and humans in China (Liu et al., 2016). Since then, this gene has been increasingly reported in *Escherichia coli, Klebsiella pneumonia*, and *Salmonella* species (Castanheira et al., 2016; Falgenhauer et al., 2016; Quan et al., 2017; Lu et al., 2019). The *mcr*-*1* gene carrying *S. typhimurium* has been reported in humans, food-producing animals and their surrounding environment, raw meat samples in many countries, and ready-to-eat (RTE) meat products in China (Yang et al., 2016; Litrup et al., 2017; Saavedra et al., 2017; Yi et al., 2017; Carfora et al., 2018; Wang et al., 2018; Rau et al., 2020; Moon et al., 2021). Some of these reported strains were observed to also carry plasmid-mediated FQs resistance genes or extended-spectrum beta-lactamases (ESBLs). However, the co-occurrence frequency of plasmid-mediated *mcr*-*1*, ESBLs, and FQs genes in *S. typhimurium* remains very low, having only been sporadically reported in retail pork samples (Hu et al., 2019) and human isolates (Lu et al., 2019; Luo et al., 2020), and has not previously been reported from RTE food product sources. The emergence and co-transfer of plasmid-mediated *mcr*-*1*, ESBLs, and FQs genes among foodborne *Salmonella* might compromise the effectiveness of current antimicrobial strategies, which constitute a serious public risk for humans (Falgenhauer et al., 2016).

Pork consumption has been reported as a likely source of contamination for humans to acquire *mcr*-*1*, ESBLs, and FQs genes carrying *S*. *typhimurium* strains (Lu et al., 2019). In this study, we characterized 11 *mcr-1*-carrying *S*. *typhimurium* isolates from raw pork and RTE pork products and tracked their source to gain insight into their public health impact.

## Materials and Methods

### Strains Isolation and Identification

During our routine surveillance of foodborne pathogens from various food products during 2016–2017 in Guangdong, China, 11 *S*. *typhimurium* isolates resistant to colistin and carrying the *mcr*-*1* gene were recovered. One of the isolates (named GSJ/2017-*Sal.-*008, hereafter 17Sal008) was isolated from a retail RTE dumpling with pork and cabbage stuffing in Guangzhou in 2017, while the remaining 10 isolates were collected from raw pork products from retail markets in Guangzhou and Heyuan city in 2016 (Supplementary Table 1). The isolates were identified by biochemical confirmation using API 20E test identification test strips (bioMérieux, France), as well as amplification of the *invA* gene by PCR (Bai et al., 2016). The serotype was determined by the slide agglutination test, using *Salmonella* antisera (SSI Diagnostica, Denmark) according to the White-Kauffmann-Le Minor scheme.

The isolates were routinely grown in Luria-Bertani (LB; Guangdong Huankai Microbial Sci & Tech, Guangzhou, China) broth or on LB agar plates at 37°C for 12–24 h.

### Antibiotic Susceptibility Testing

The susceptibility of the *Salmonella* isolates to a panel of antimicrobial drugs (Hangzhou Microbial Reagent Co., Ltd., China), such as amikacin, ampicillin, ampicillin-sulbactam sodium, amoxicillin clavulanic acid, aztreonam, ciprofloxacin, cefazolin (1st generation), cefoxitin (2nd generation), cefuroxime (2nd generation), cefotaxime (3rd generation) and ceftazidime (3rd generation), cefepime (4th generation), chloramphenicol, doxycycline, ertapenam, fosfomycin, gentamicin, imipenem, meropenem, nalidixic acid, netilmicin, peracillin, tetracycline, trimethoprim/sulfamethoxazole, tigecycline, and tobramycin, was determined by disk diffusion antibiotic susceptibility testing (CLSI, 2017). Minimal inhibitory concentrations (MICs) to polymyxin B/colistin, ciprofloxacin, and cefotaxime (Sigma-Aldrich, St. Louis, MO) were determined by broth microdilution (CLSI, 2017). Results were interpreted according to the CLSI breakpoints; i.e., *Salmonella* isolates with MICs of colistin ≤2 μg/ml were categorized as susceptible, and those with MICs ≥4 μg/ml were recorded as resistant. For cefotaxime, isolates with MICs of ≤1 μg/ml were considered susceptible, and those with MICs ≥4 μg/ml were categorized as resistant. For ciprofloxacin, isolates with MICs ≤0.06 μg/ml were considered susceptible, while those with MICs of ≥1 μg/ml were considered resistant. A tentative ESBL production phenotype was confirmed by a double-disk test comparing the zone diameters between ceftazidime (30 μg) and cefotaxime-clavulanic acid (30/10 μg) disks and between cefotaxime (30 μg) and ceftazidime-clavulanic acid (30/10 μg) disks (CLSI, 2017). The reference strain *E. coli* ATCC 25922 served as quality control.

### Whole-Genome Sequencing and Annotation

The genomic DNA of the isolates was extracted using a commercial DNA extraction kit (Magen, Guangzhou, China) following the manufacturer's recommendations. The whole genome of each isolate was sequenced on Illumina Hiseq×10 with 150 bp paired-end reads (MajorBio Co., Shanghai, China). Illumina sequencing generated at least 368x sequence coverage depth which was sufficient to allow further analysis (Supplementary Table 2). The genome of 17Sal008 was further sequenced on MinION (Oxford Nanopore, Oxford, United Kingdom). For the MinION platform, the library was prepared using the ONT 1D ligation sequencing kit (SQK-LSK109) with the native barcoding expansion kit (EXP-NBD104). The genome was assembled using a combination of short- and long-reads by SPAdes V3.14.0 (Bankevich et al., 2012) and Unicycler hybrid assembler V0.4.8 (Wick et al., 2017), and annotated by Prokka V1.14.6 (Seemann, 2014).

Clonal analysis was assessed by MLST 2.0 (http://mlst.warwick.ac.uk/mlst/dbs/Senterica). The presence of acquired antibiotic resistance genes and mutations in the quinolone resistance-determining regions (*gyrA, gyrB, parC*, and *parE*) was assessed by ResFinder V4.1 (Zankari et al., 2012), and was further determined by BLASTn (http://blast.ncbi.nlm.nih.gov/Blast.cgi). The plasmid was predicted by PlasmidFinder V2.0.1 (Carattoli et al., 2014). The plasmid of 17Sal008 was compared with the most closely related plasmids using BLASTn (http://blast.ncbi.nlm.nih.gov/Blast.cgi) and BLAST Ring Image Generator (BRIG) (Alikhan et al., 2011).

### Phylogenetic Analysis of the Genomic Sequences

To assess the relatedness of foodborne *S. typhimurium* isolates recovered in this study with other *S. typhimurium* strains from different sources in China, we retrieved all 83 genome sequences of *S. typhimurium* that have been released from EnteroBase databases and performed core-genome Multi Locus Sequence Typing (cgMLST) (cgMLST scheme available on EnteroBase, https://enterobase.warwick.ac.uk, accessed on 9 March 2022). Similar but non-identical strains [strains showing different core genome Sequence Types (cgST)] were identified in EnteroBase by using the hierarchical clustering method (HierCC) that allows for grouping of strains into hierarchical clusters (HCs) that can differ up to a specified and fixed number of cgMLST alleles. This number is indicated by the suffix following “HC” (e.g., HC5 for 5 cgMLST allelic differences). Isolates belonging to the same HC10 cluster were considered a possible epidemiologically linked, and isolates belonging to the same HC5 cluster were considered highly probably epidemiological linked (Bonifait et al., 2021).

To assess the genetic relationship between strains, a minimum-spanning tree was created from cgMLST allelic differences in EnteroBase using GrapeTree with the RapidNJ algorithm (Zhou et al., 2020). The assembly sequences are publicly available from EnteroBase; their accession numbers (barcodes) are listed in Supplementary Table 1.

### Conjugation

Conjugation was conducted by solid mating on a filter (Whatman, Maidstone, UK) by using sodium azide-resistant *E. coli* J53 as a recipient, and selection of transconjugants on LB agar containing 150 μg/ml sodium azide and 16 μg/ml cefotaxime, as previously described (Li et al., 2021). The transfer of plasmid to transconjugants was confirmed by PCR targeting the *mcr*-*1* gene with primer mcr-1-F (5′- ATGATGCAGCATACTTCTGTG-3′) and mcr-1-R (5′-TCAGCGGATGAATGCGGTG-3′) (Luo et al., 2017), and further sequenced the plasmid DNA extracted from selected transconjugants on Illumina Hiseq platform (MajorBio Co., Shanghai, China).

### Nucleotide Sequence Accession Number

The raw sequence data of all 11 isolates were deposited in the Enterobase database under the barcode numbers: SAL_LB2715AA to SAL_LB2720AA. The assembly genome sequence of *S. typhimurium* 17Sal008 was deposited in the Nucleotide database under the accession number: CP050130 and CP050131.

## Results

### Identification of *Salmonella*

The obtained isolates were confirmed as *S. enterica* serovar Typhimurium by biochemical confirmation, 16S rRNA gene sequencing, serotyping, and whole-genome sequencing. Multi-locus sequence typing analysis showed that all isolates belong to sequence type 34 (ST34).

### Antibiotic Susceptibility and Antibiotic Resistance Determinants

**Figure 1:**
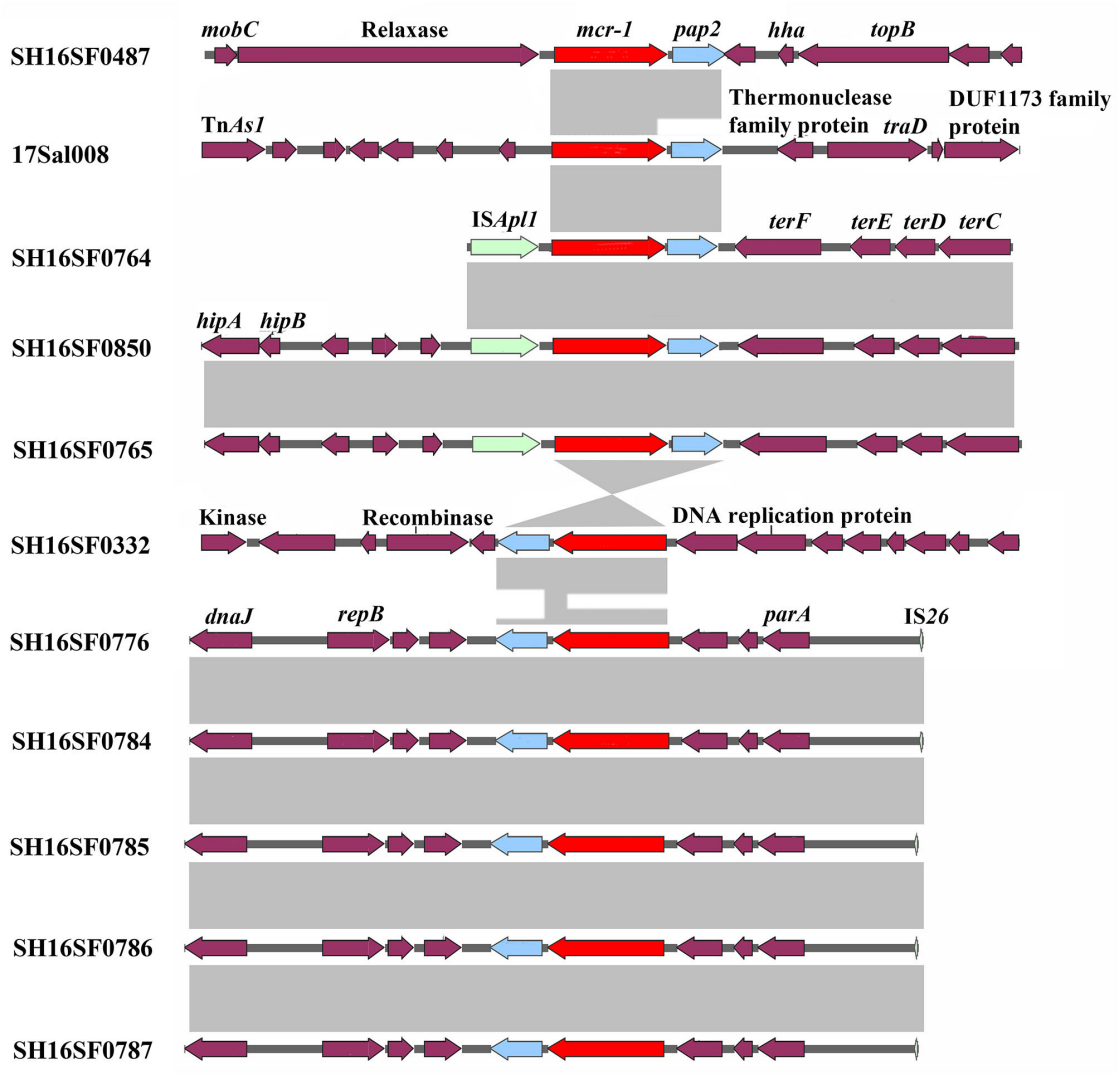
Genetic comparison of *mcr-1* gene contexts of 11 foodborne *Salmonella typhimurium*. Light gray shading denotes homology regions.

All isolates were multi-drug resistant (MDR), exhibiting resistance to 5–10 antibiotic classes and were confirmed to carry 6–24 resistance genes by Resfinder (Table 1). All isolates were resistant to colistin with a MIC value of 4 μg/ml and carried the *mcr-1* gene (Table 1). The *mcr*-*1* gene was in the most conserved structure *mcr*-*1*-*orf* (the *orf* encodes the putative PAP family transmembrane protein) in eight isolates, and in the mobile structure IS*Apl1*-*mcr*-*1*-*orf* in three isolates (Figure 1).

**Table 1:** The antibiotic resistance profiles of *Salmonella typhimurium* isolates and the selected transformant of 17Sal008 (17Sal008T).

**Strains**	**lnc group**	**MIC (mg/L)**				**Resistance determinants**		**Other resistance genes**	**Antibiotic resistance^**b**^**
		**PB**	**CIP**	**CTX**	**PB**	**CIP**	**ESBLs**		
SH16SF0332	IncHI2 IncN	4	8	4	*mcr-1*	*oqxA, oqxB, qnrS2*	–	*arr-3, aac(6')-Ib-cr, aadA1, aadA2, aph(3')-Ia*, *bla*_OXA−1_, *catB3, cmlA1, dfrA12, floR, mef(B), mph(A), sul1, sul3, tet(A), tet(B), tet(M)*	TOB, FEP, CHL, AMP, CIP, PB, SXT, NAL, TET
SH16SF0487	IncI2	4	<0.25	1	*mcr-1*	*-*	–	*aph(3”)-Ib, aph(6)-Id*, *bla*_TEM−1β_, *tet(B), sul2*	TOB, AMP, PB, SXT, TET
SH16SF0764	IncHI2	4	<0.25	<0.25	*mcr-1*	*oqxA, oqxB*	–	*aadA1, aadA2, aph(3”)-Ib, aph(6)-Id*, *bla*_TEM−1β_, *cmlA1, sul2, sul3, tet(B)*	TOB, CHL, AMP, CIP, PB, SXT, NAL, TET
SH16SF0765	IncHI2	4	<0.25	4	*mcr-1*	*oqxA, oqxB*	–	*aadA1, aadA2, aph(3”)-Ib, aph(6)-Id*, *bla*_TEM−1β_, *cmlA1, sul2, sul3, tet(B)*	PB, TOB, CHL, AMP, CIP, SXT, NAL, TET
SH16SF0776	IncFII IncHI2 IncX4	4	4	4	*mcr-1*	*oqxA, oqxB*	–	*arr-3, aac(3)-IV, aac(6')-Ib-cr, aadA1, aph(3')-Ia, aph(3”)-Ib, aph(4)-Ia, aph(6)-Id*, *bla*_OXA−1_, *catB3, cmlA1, floR, mph(A), sul1, sul2, sul3, tet(A), tet(B)*	TOB, FEP, CHL, AMP, CIP, PB, SXT, NAL, TET, GEN
SH16SF0784	IncFII IncHI2 IncX4	4	4	8	*mcr-1*	*oqxA, oqxB*	–	*arr-3, aac(3)-IV, aac(6')-Ib-cr, aadA1, aph(3')-Ia, aph(3”)-Ib, aph(4)-Ia, aph(6)-Id*, *bla*_OXA−1_, *catB3, cmlA1, floR, sul1, sul2, sul3, tet(A), tet(B)*	TOB, FEP, CHL, AMP, CIP, PB, SXT, NAL, TET, GEN
SH16SF0785	IncFII IncHI IncX4	4	4	8	*mcr-1*	*oqxA, oqxB*	*-*	*arr-3, aac(3)-IV, aac(6')-Ib-cr, aadA1, aph(3')-Ia, aph(3”)-Ib, aph(4)-Ia, aph(6)-Id*, *bla*_OXA−1_, *catB3, cmlA1, floR, mph(A), sul1, sul2, sul3, tet(A), tet(B)*	TOB, FEP, CHL, AMP, CIP, PB, SXT, NAL, TET, GEN
SH16SF0786	IncFII IncHI IncX4	4	4	16	*mcr-1*	–	–	*arr-3, aac(3)-IV, aac(6')-Ib-cr, aph(3”)-Ib, aph(4)-Ia, aph(6)-Id*, *bla*_OXA−1_, *catB3, floR, mph(A), sul1, sul2, tet(A), tet(B)*	TOB, FEP, CHL, AMP, CIP, PB, SXT, TET, GEN
SH16SF0787	IncFII IncHI IncX4	4	1	32	*mcr-1*	–	–	*arr-3, aac(3)-IV, aac(6')-Ib-cr, aph(4)-Ia, aph(6)-Id*, *bla*_OXA−1_, *catB3, mph(A), sul1, sul2, tet(A), tet(B)*	TOB, FEP, CHL, AMP, CIP, PB, SXT, TET, GEN
SH16SF0850	IncHI2	4	8	256	*mcr-1*	*oqxA, oqxB*	*bla*_CTX−M−14_	*aadA1, aph(3')-Ia, cmlA1, fosA3, sul1, sul3*	TOB, FEP, CHL, AMP, CTX, CIP, PB, SXT, NAL, CAZ
17Sal008	IncHI2	4	4	128	*mcr-1*	*oqxA, oqxB, qnrS2*	*bla*_CTX−M−14_	*arr-3, aph(3')-Ia, aadA1, aadA2, aac(6')-Ib-cr, aac(3)-IV, aph(4)-Ia, aph(3”)-Ib, aph(6)-Id*, *bla*_OXA−1_, *cmlA1, catB3, dfrA12, floR, sul1, sul2, sul3, tet(A), tet(B)*	TOB, FEP, CHL, AMP, CTX, CIP, PB, SXT, NAL, TET, CAZ, GEN
17Sal008T	IncHI2	4	4	128	*mcr-1*	*oqxA, oqxB, qnrS2*	*bla*_CTX−M−14_	*arr-3, aph(3')-Ia, aadA1, aadA2, aac(6')-Ib-cr, aac(3)-IV, aph(4)-Ia*, *bla*_OXA−1_, *dfrA12, cmlA1, catB3, floR, sul1, sul2, sul3, tet(A)*	TOB, FEP, CHL, AMP, CTX, CIP, PB, SXT, NAL, TET, CAZ, GEN
*E. coli* J53		0.5	<0.5	<0.25					

*^a^TOB, tobramycin; FEP, cefepime; CHL, chloramphenicol; AMP, ampicillin; CIP, ciprofloxacin; PB, polymyxin B; SXT, trimethoprim/sulfamethoxazole; NAL, nalidixic acid; TET, tetracycline; GEN, gentamicin; CAZ, ceftazidime*.

Nine isolates were resistant to cefotaxime with an MIC value of 4–256 μg/ml. Of them, two isolates produced ESBLs conferring high resistance levels to cefotaxime (with MIC values of 128 and 256 μg/ml, respectively), and were found to carry the *bla*_CTX−M−14_ gene. Seven isolates exhibited lower cefotaxime resistance with MIC values ranging from 4 to 32 μg/ml (Table 1).

Eight isolates were resistant to ciprofloxacin (with MIC values of 1–8 μg/ml, respectively), and seven of them harbored *oqxAB* and/or *qnrS2* (Table 1). No FQs resistance genes were identified in two isolates (SH16SF0786 and SH16SF0787). Mutations were not identified in the quinolone resistance-determining regions (*gyrA, gyrB, parC*, and *parE*) in all isolates.

Phenotypically, eight isolates were co-resistant to colistin, ciprofloxacin, and cefotaxime (Table 1). Among these isolates, one pork isolate SH16SF0850 co-harbored *mcr*-*1*, *bla*_CTX−M−14_, and *oqxAB*, and the RTE pork isolate 17Sal008 co-harbored *mcr*-*1*, *bla*_CTX−M−14_, *oqxAB*, and *qnrS2* genes (Table 1). In addition, all isolates were predicted to contain plasmids with replicon types IncI2, IncHI2, IncN, IncFII, and IncX4 (Table 1). Notably, both isolates SH16SF0850 and 17Sal008 carried *mcr*-*1*, *bla*_CTX−M−14_, *oqxAB*, and/or *qnrS2* genes contained IncHI2 plasmid.

### Phylogenetic Analysis and Genomic Comparisons

**Figure 2:**
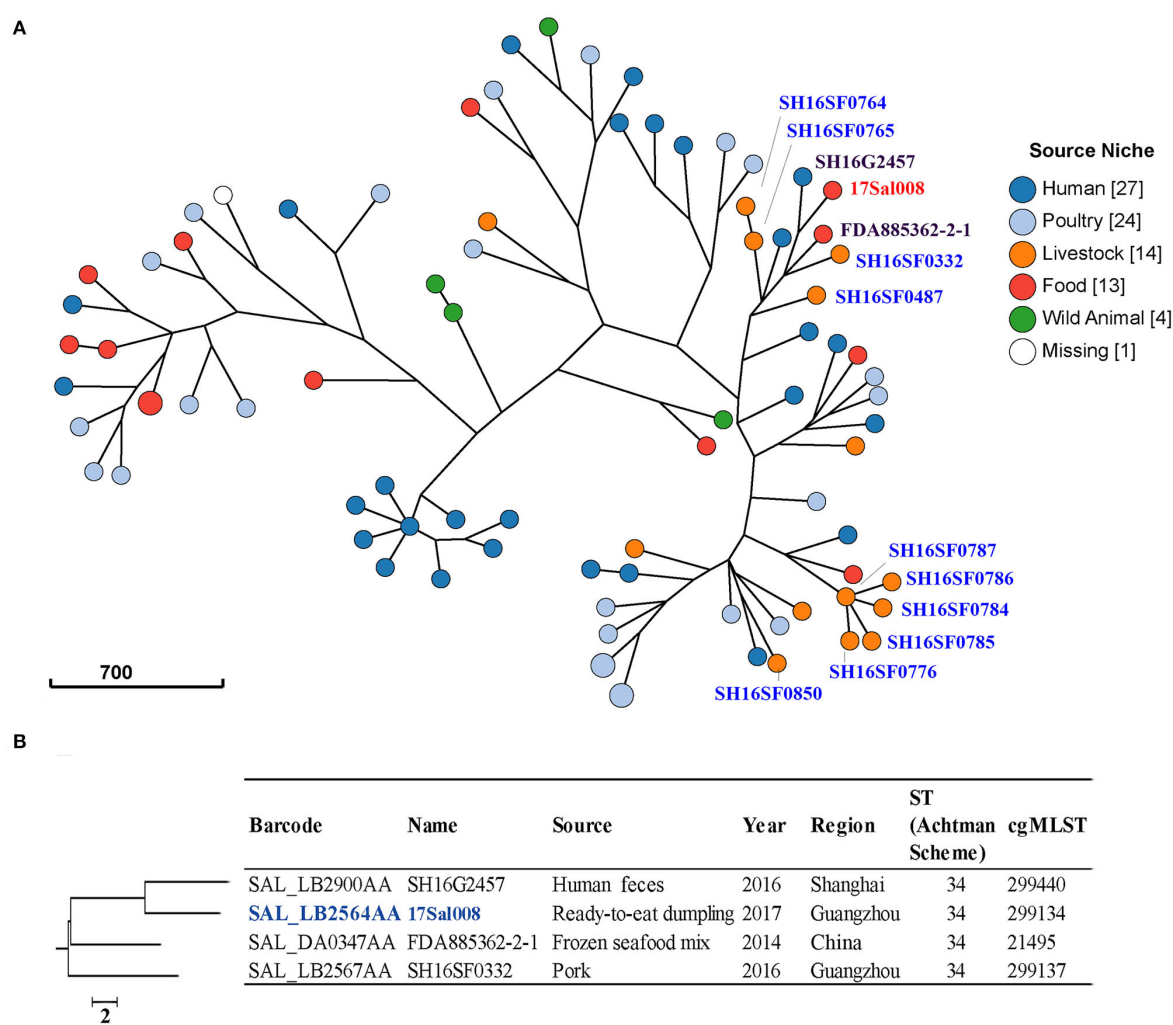
Phylogenetic analysis of 83 *S. typhimurium* isolates from different sources in China. **(A)** A minimum-spanning tree based on core-genome Multi Locus Sequence Typing (cgMLST) analysis using a log depiction of branch length. The position of 17Sal008 isolate is indicated in red font and pork isolates in blue font. Each circle represents a cgMLST group and the size of the circle is proportional to the number of isolates in that group. **(B)** Detailed information of strains in the branch contained 17Sal008. The 17Sal008 isolate is marked blue.

In this study, we found several clusters among the 83 *S. typhimurium* isolates from different sources in China (Figure 2A). These isolates belonged to six classical MLST types, with the most frequent being ST19 (45.8%) and ST34 (45.8%) (Supplementary Figure 1). cgMLST and phylogenetic analysis showed that all isolates harbored a unique cgST profile (Supplementary Table 1).

For the pork isolates, five of them (SH16SF0784, SH16SF0785, SH16SF0786, SH16SF0787, and SH16SF0776) collected in the same batch from Heyuan city were clustered together, and they belonged to the same HC5 cluster (Figure 2A, Supplementary Table 1), indicating they were highly probably epidemiological linked. However, another two isolates (SH16SF0764 and SH16SF0765) from this batch were not linked with these five isolates, as indicated by different numbers at the HC10 cluster. Instead, the two isolates (SH16SF0764 and SH16SF0765) together with SH16SF0332 and SH16SF0487 were clustered with human isolate SH16G2457 (Barcode: SAL_LB2900AA), seafood isolate FDA885362-2-1 (Barcode: SAL_DA0347AA), and RTE pork isolate 17Sal008 (Figure 2A). However, no epidemiological links were observed among them (Supplementary Table 1). Specifically, for RTE pork isolate 17Sal008, cgMLST results differentiated it with closely related human isolate SH16G2457 and seafood isolate FDA885362-2-1, as well as pork isolate SH16SF0332 up to HC10 level (a maximum of 10 cgMLST allelic variations) (Figure 2B, Supplementary Table S3). Therefore, it did not allow for inferring any epidemiological links between RTE pork isolate 17Sal008 with other strains in China.

In addition, no epidemiological links were identified in isolates from the same region but in different batches, such as pork isolates SH16SF0332, SH16SF0487, and SH16SF0850 (Figure 2A, Supplementary Table 1).

### Comparative Analysis of Plasmid and Genetic Contexts Analysis

**Figure 3:**
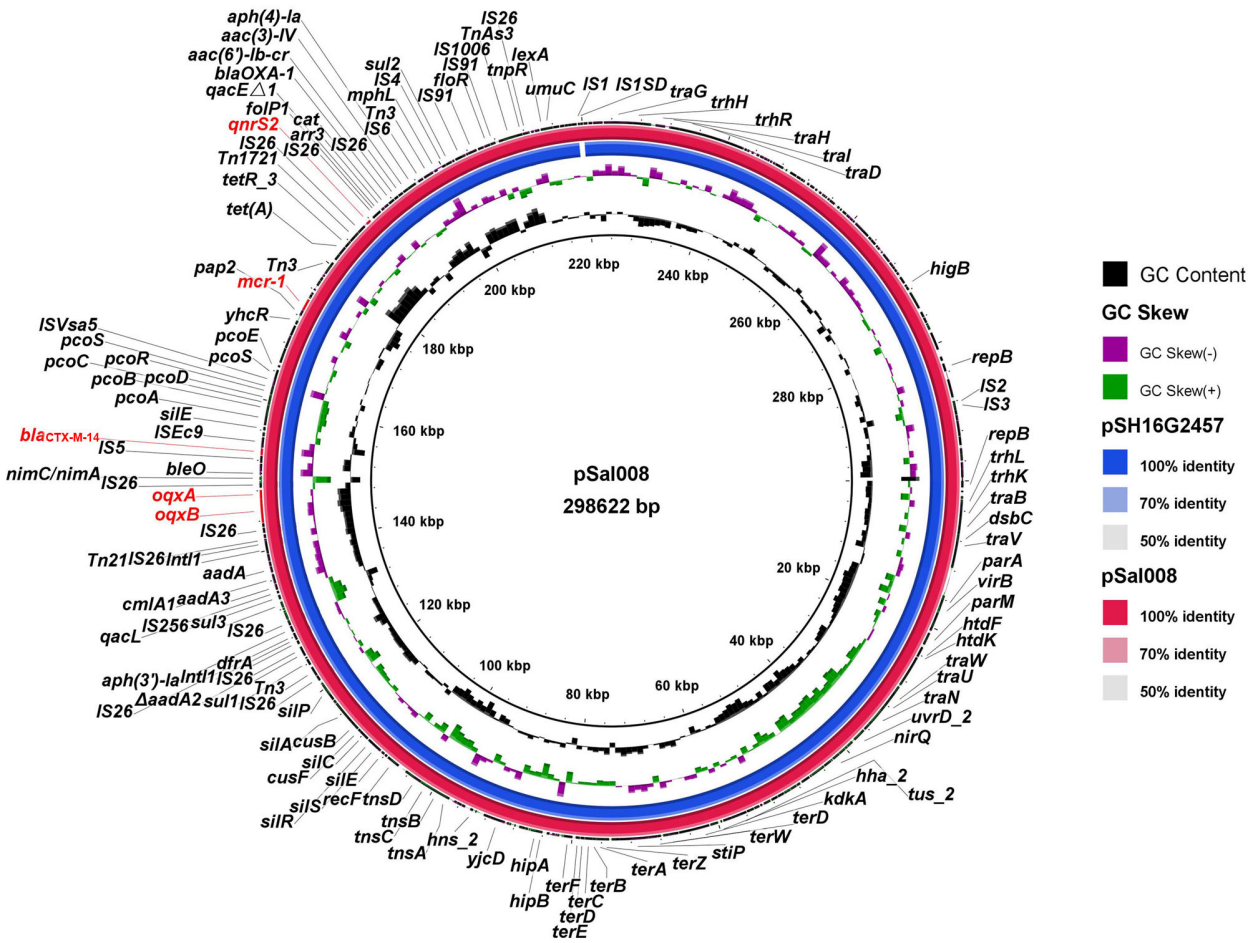
Sequence comparison of plasmid pSal008 with pSH16G2457 (GenBank no. MH522421.1) from a clinical *S. typhimurium* strain in BLAST Ring Image Generator (BRIG). Reference plasmid pSH16G2457 is indicated in blue in the inner circles.

As *S*. *typhimurium* 17Sal008 was an MDR and an isolate identified from RTE pork product that was co-resistant to colistin, 3GCs, and FQs and harbored *mcr*-*1*, *bla*_CTX−M−14_, *qnrS2*, and *oqxAB* genes which have not been reported previously, we further revealed the genetic contexts of MDR genes by a combination of short- and long-read sequencing (HiSeq and MinION). *S. typhimurium* 17Sal008 contained a circular plasmid, denoted as pSal008. The pSal008 is a 298,622 bp IncHI2 plasmid, with 374 predicated CDSs and an average GC content of 46.9%. The pSal008 co-harbored 21 antibiotic resistance genes encoding resistance to aminoglycoside [*aac(3)*-*IV, aac(6')*-*Ib*-*cr, aadA1, aadA2, aph(3')*-*Ia* and *aph(4)*-*Ia*], colistin (*MCR*-*1*), β-lactam (*bla*_OXA−1_, *bla*_CTX−M−14_), fluoroquinolone (*oqxAB, qnrS2*), phenicol (*catB3, floR*, and *cmlA1*), rifampicin (*arr*-*3*), sulfonamide (*sul1, sul2*, and *sul3)*, tetracycline [*tet(A)*], and trimethoprim (*dfrA12*). In addition, anticancer agents-bleomycin resistance encoding gene *bleO* along with the 5-nitroimidazole-based (5-Ni) antimicrobial resistance-encoding gene *nimC*/*nimA* were identified. The plasmid also harbors quaternary ammonium resistance genes (*qacL* and *qacE*Δ*1*) and a large number of metal tolerance genes, such as efflux systems to detoxify copper (*pcoABCDRSE, cusF*, and *cusB*), silver (*silACEPRS*), as well as tellurite resistance systems (*terABCDEFWZ*). Pathogenicity-related virulence gene *virB* and HigB/HigA toxin/antitoxin system were also found on the plasmid. Transposons, such as IS*26*, were found to be abundant on the plasmid. In addition, two copies of Class I integrase were located on the plasmid (Figure 3).

BLASTn comparison of the entire plasmid sequence to microbial sequences in GenBank indicated that it was most closely related to pSH16G2457 (GenBank no. MH522421.1) from a *S. typhimurium* strain isolated from a 1-year-old diarrheal outpatients in Shanghai, China in 2016 (Figure 3), with 99.98% nucleotide identity and 99% sequence coverage.

The *mcr-1* gene in pSal008 was in the *mcr-1*-*orf* structure. The *oqxAB, qnrS2*, and *bla*_CTX−M−14_ were found to be located in composite transposons, IS*26*-*orf*-*oqxA*-*oqxB*-IS*26*, IS*5*-*bla*_CTX−M−14_-*orf*-IS*Ec9*, and IS*26*-*orf*-*qnrS2*-IS*26*-*folP1*-*qacE*Δ*1*-*arr3*-*cat*-*bla*_OXA−1_-*aac(6')-Ib-cr-*IS*26*, respectively. The latter composite transposon contained an MDR gene cluster. BLASTn comparison of the composite transposons showed that they were widely distributed in chromosomal or plasmids of various *Enterobacteriaceae* species (data not shown).

### Horizontal Transfer of the pSal008

PCR and sequencing results confirmed the successful transfer of the plasmid pSal008 to a plasmid-free recipient, *E. coli* J53. Antimicrobial susceptibility testing revealed the acquisition of the plasmid by *E. coli* caused an 8-fold increase in the MIC value of polymyxin B, an 8-fold increase for ciprofloxacin, and a 512-fold increase for cefotaxime (Table 1).

## Discussion

The prophylactic use of colistin as a feed additive before 2016 has resulted in a significant increase in the rate of colistin resistance among organisms isolated from livestock and poultry farms in China (Shen et al., 2016). Food-producing animals have been considered a major reservoir of *mcr*-*1*-carrying bacteria (Liu et al., 2016; Yi et al., 2017; Moon et al., 2021; Sevilla et al., 2021; Timmermans et al., 2021). In this regard, pork has been considered one of the major contamination sources of *mcr-1*-harboring *Salmonella* (Hu et al., 2019; Lu et al., 2019; Elbediwi et al., 2020). Additional whole genomic characterization of *mcr-1*-harboring *Salmonella* from pork and pork products is required to fully understand the mechanism of their transmission and control their spread along the food chain. The *mcr*-*1* gene positive *S*. *typhimurium* ST34 strains were considered the main serotype contributing to the spread of the *mcr*-*1* gene among food-producing animals in China (Wong et al., 2013; Sun et al., 2014; Yi et al., 2017). In this study, we characterized 11 *mcr*-*1*-harboring *S*. *typhimurium* isolates recovered from retail pork and RTE pork products.

All of the isolates were MDR and contained multiple resistance genes. The colistin resistance was predicted to be encoded by the *mcr-1* gene. Different resistance levels were observed in 3GCs resistant strains. The strains exhibiting high-level resistance to cefotaxime were predicted to be associated with *bla*_CTX−M−14_. Several strains were found to exhibit a low resistance level to cefotaxime without corresponding resistance genes, which might be explained by other mechanisms, such as efflux pumps (Jacoby, 2009). All ciprofloxacin-resistant strains were low-level resistant, which may be contributed to the combination of efflux pumps (*oqxAB*) and the presence of plasmid-mediated quinolone resistance (PMQR) determinant (*qnrS*) (Lin et al., 2015). In addition, no PMQRs and target mutations were observed in two ciprofloxacin-resistant isolates, which leads to the mechanisms to be further explored.

Importantly, most of these isolates were co-resistant to the front-line antibiotics, colistin, 3GCs, and FQs. Two of these isolates were identified to harbor plasmid-mediated *mcr*-*1*, ESBLs, and FQs genes. Co-occurrence of plasmid-mediated *mcr*-*1*, ESBLs, and FQs genes in *Salmonella* has only been sporadically reported (Hu et al., 2019; Lu et al., 2019; Luo et al., 2020). A large-scale epidemiological survey of 2,555 *Salmonella* isolates cultured from foods in China found only one isolate of *S*. London from raw pork that co-harbored plasmid-mediated *mcr*-*1*, *bla*_CTX−M−55_, and *qnrS1* genes (Hu et al., 2019). Three out of 12,053 *Salmonella* isolates from diarrheal outpatients in China were confirmed to co-harbor *mcr*-*1*, ESBLs, and *qnrS* genes (Lu et al., 2019). One *S. typhimurium* isolate, collected from 280 bloodstream and 110 intestinal infection samples from inpatients in 15 provinces from 2014 to 2017, was confirmed to carry *mcr*-*1*, *bla*_CTX−M−14_, *oqxAB*, and *qnrS1* genes (Luo et al., 2020). To the best of our knowledge, *S. typhimurium* ST34 isolate from an RTE food product harboring plasmid-mediated *mcr*-*1*, *bla*_CTX−M−14_, *oqxAB*, and *qnrS2* genes has not been reported previously. Despite the frequency of co-occurrence of *mcr*-*1*, ESBLs, and FQs genes in *Salmonella* remains very low, the emergence of plasmid-mediated *mcr*-*1*, *bla*_CTX−M−14_, *oqxAB*, and *qnrS2* genes in RTE pork product raises serious concern and should be further investigated, as they may transfer to a human directly.

The *mcr-1* gene was found to be more often inserted into the most conserved structure *mcr-1*-*orf* in pork and RTE pork isolates, suggesting it was stabilized and plasmids might be the more efficient vehicle for its disseminating. In RTE pork isolate 17Sal008, the *bla*_CTX−M−14_, *oqxAB*, and *qnrS2* genes were located in composite transposons, which have been identified in plasmids of various *Enterobacteriaceae* species, indicating the acquisition of these genes by the plasmid pSal008 and the transferability of these genes among different bacteria species.

The IncHI2 type plasmid pSal008 identified in this study was observed to be transferable to *E. coli*. The highly similar pSH16G2457 has been confirmed to be capable of transferring from *S*. *typhimurium* strain to *S. typhi* and *K. pneumoniae*, and from *E. coli* to *Salmonella spp*. previously (Lu et al., 2019). Interestingly, phylogenetic and cgMLST analysis indicated that the host of pSal008 and the highly similar pSH16G2457 had no possible epidemiological links, which suggests the transfer of the plasmid among different *S*. *typhimurium* strains. These results together indicated that this plasmid is highly transferable and might contribute to the development of co-resistance to colistin, 3GCs, and FQs, which will compromise the effectiveness of current antimicrobial strategies and impose a therapeutic challenge.

Phylogenetic and cgMLST analysis showed that several *mcr-1* carrying pork *S. typhimurium* isolates from the same region and the same batch were highly probably epidemiological linked, suggesting they might come from the same source. Moreover, different *mcr-1* carrying *S. typhimurium* strains were present in pork regardless of the same or different regions. In addition, no possible epidemiological links were found in the colistin, 3GC, and FQs co-resistant RTE pork *S. typhimurium* 17Sal008 with isolates from different sources in China. Thus, the source of this isolate remains obscure. We suggest a continuous surveillance program be conducted to monitor the epidemic trends of *Salmonella* with colistin, 3GC, and FQs resistance in animal products, food, the community, and hospitals to help put forward effective control measures.

## Conclusion

In summary, our study revealed that the *mcr-1* harboring *S. typhimurium* from raw pork and RTE pork products was all MDR, contained multiple genes, and some of them were highly probably epidemiological linked, indicating that pork and pork products were potential reservoirs of *mcr-1*-harboring *S. typhimurium*. To the best of our knowledge, we describe the first *S. typhimurium* ST34 isolate obtained from an RTE food product co-harboring plasmid-mediated *mcr*-*1*, *bla*_CTX−M−14_, *oqxAB*, and *qnrS2* genes. The transmission of this plasmid may accelerate the development and dissemination of isolates co-resistant to colistin, 3GCs, and FQs that are front-line drugs of choice for treating severe *Salmonella* infections. Thus, sustained surveillance needs to be conducted to monitor the epidemic trends of *Salmonella* with plasmid-mediated *mcr*-*1*, ESBLs, and FQs genes in animal products and RTE food products to prevent their transmission along the food chain.

## Data Availability Statement

The datasets presented in this study can be found in online repositories. The names of the repository/repositories and accession number(s) can be found in the article/Supplementary Material.

## Author Contributions

LL performed the experiment and wrote the manuscript. HM conceptualized and designed the study. XW involved in data analysis. RO revised the manuscript. JX and XX provided the strains. CW and LS contributed reagents, materials, and analysis tools. All authors contributed to the article and approved the submitted version.

## Funding

This work was supported by the National Natural Science Foundation of China (Grant Nos. 31901789 and 32001796), the Natural Science Foundation of Guangdong Province (Grant Nos. 2022A1515011685 and 2020A1515010218), and the Basic Research Project of Guangzhou (Grant No. 202002030145).

## Conflict of Interest

CW was employed by Shandong New Hope Liuhe Group Ltd., Qingdao, China. The remaining authors declare that the research was conducted in the absence of any commercial or financial relationships that could be construed as a potential conflict of interest.

## Publisher's Note

All claims expressed in this article are solely those of the authors and do not necessarily represent those of their affiliated organizations, or those of the publisher, the editors and the reviewers. Any product that may be evaluated in this article, or claim that may be made by its manufacturer, is not guaranteed or endorsed by the publisher.
