# Supplementary material for: Genomic Characterization of mcr-1-Carrying Foodborne Salmonella enterica serovar Typhimurium and Identification of a Transferable Plasmid Carrying mcr-1, blaCTX-M-14, qnrS2, and oqxAB Genes From Ready-to-Eat Pork Product in China
Source: Front Microbiol. 2022 Jun 29;13:903268. doi: 10.3389/fmicb.2022.903268 (PMC9277226; doi:10.3389/fmicb.2022.903268)

**Supplementary data**

**Supplementary Table S2. Summary of the Illumina sequencing raw data.**

| **Name** | **Coverage** |  | **N50** | **Length** | **Contig Number (>=200 bp)** | **Low Quality Bases** | **Assembly Barcode** |
| --- | --- | --- | --- | --- | --- | --- | --- |
| 17Sal008 | 502 |  | 270592 | 5240684 | 141 | 8108 | SAL_TC5922AA_AS |
| SH16SF0332 | 378 |  | 223309 | 5232493 | 114 | 31195 | SAL_TC5925AA_AS |
| SH16SF0487 | 368 |  | 282781 | 4993151 | 80 | 7732 | SAL_TC5926AA_AS |
| SH16SF0764 | 377 |  | 223309 | 5190199 | 91 | 15376 | SAL_TC6187AA_AS |
| SH16SF0765 | 374 |  | 282781 | 5188041 | 81 | 14657 | SAL_TC6188AA_AS |
| SH16SF0784 | 408 |  | 270591 | 5239254 | 109 | 14744 | SAL_TC6202AA_AS |
| SH16SF0785 | 420 |  | 259053 | 5238798 | 108 | 14719 | SAL_TC6203AA_AS |
| SH16SF0786 | 387 |  | 270591 | 5215885 | 95 | 13444 | SAL_TC6204AA_AS |
| SH16SF0787 | 408 |  | 270591 | 5195285 | 95 | 14300 | SAL_TC6205AA_AS |
| SH16SF0850 | 429 |  | 229684 | 5084108 | 87 | 23034 | SAL_TC6206AA_AS |
| SH16SF0776 | 389 |  | 270591 | 5236089 | 106 | 17011 | SAL_TC6207AA_AS |

**Supplementary Table S3.** Sequence type (ST) and core genome MLST (cg MLST) profiles of the [*S*.](#/javascript:;) Typhimurium isolates that were most closely related to isolate 17Sal008 in phylogenetic analysis.

| **Barcode** | **Name** | **cgMLST** | **HC0 (indistinguishable)** | **HC2** | **HC5** | **HC10** | **HC20** | **HC50** | **HC**  **100** | **HC200** | **HC400** | **HC900 (ceBG)** | **HC2000 (Super-lineage)** | **HC**  **2600** | **HC**  **2850 (subsp.)** |
| --- | --- | --- | --- | --- | --- | --- | --- | --- | --- | --- | --- | --- | --- | --- | --- |
| SAL_LB2900AA | SH16G2457 | 299440 | 299440 | 299440 | 299440 | 299440 | 2 | 2 | 2 | 2 | 2 | 2 | 2 | 2 | 2 |
| **SAL_LB2564AA** | **17Sal008** | 299134 | 299134 | 299134 | 299134 | 299134 | 2 | 2 | 2 | 2 | 2 | 2 | 2 | 2 | 2 |
| SAL_DA0347AA | FDA885362-2-1 | 21495 | 21495 | 21495 | 21495 | 21495 | 2 | 2 | 2 | 2 | 2 | 2 | 2 | 2 | 2 |
| SAL_LB2567AA | SH16SF0332 | 299137 | 299137 | 299137 | 299137 | 2 | 2 | 2 | 2 | 2 | 2 | 2 | 2 | 2 | 2 |

**Supplementary Figure S1.** Distribution of MLST types among 83 [*S*.](#/javascript:;) Typhimurium strains.


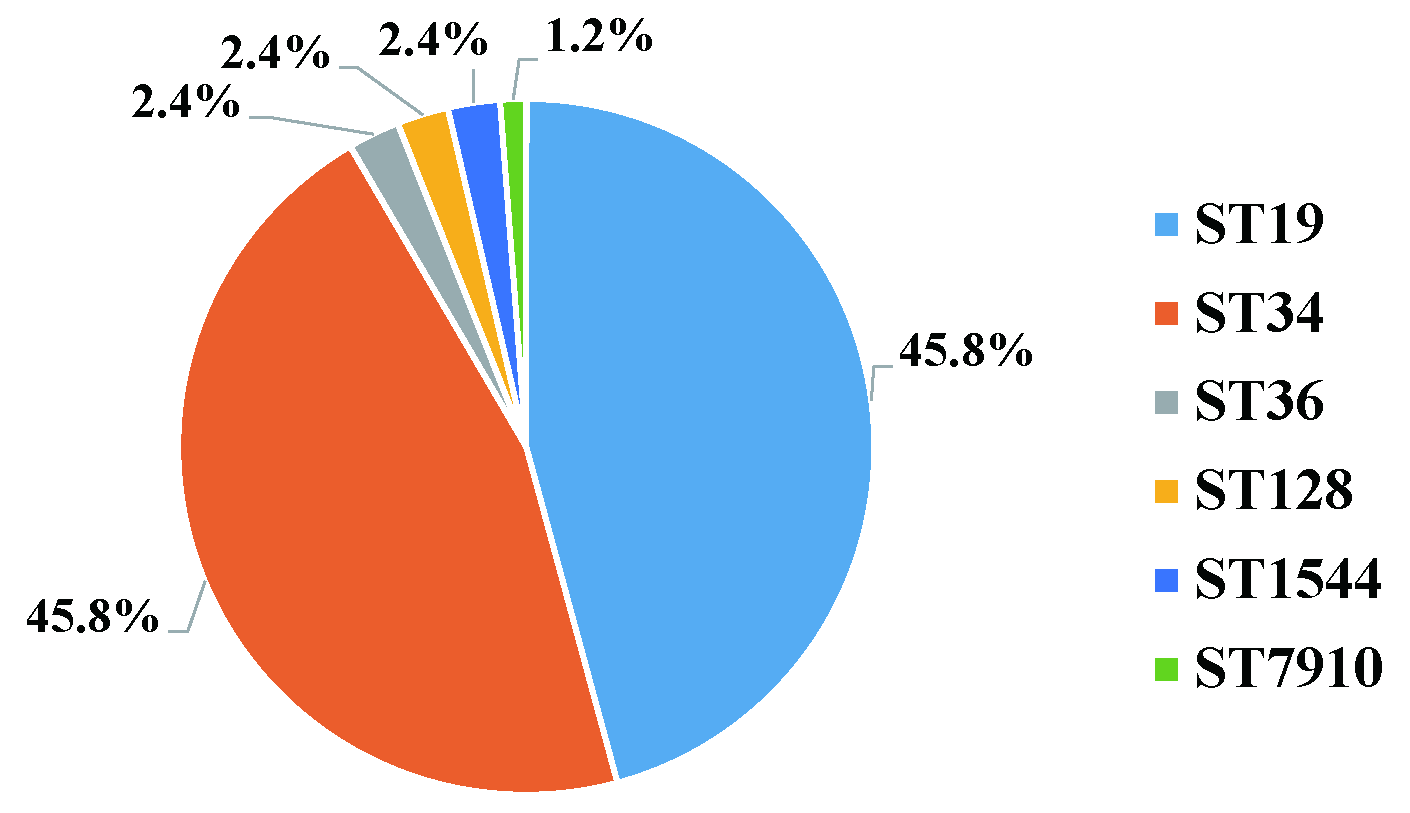

Supplement: Supplementary file 2 [file Data_Sheet_1.DOCX]
